# Exercise therapy of mild cognitive impairment: EEG could enhance efficiency

**DOI:** 10.3389/fnagi.2024.1373273

**Published:** 2024-04-10

**Authors:** Xianglong Wan, Yifan Zhang, Tiange Liu, Danyang Li, Hao Yu, Dong Wen

**Affiliations:** ^1^School of Intelligence Science and Technology, University of Science and Technology Beijing, Beijing, China; ^2^Key Laboratory of Perception and Control of Intelligent Bionic Unmanned Systems, Ministry of Education, Institute of Artificial Intelligence, University of Science and Technology Beijing, Beijing, China; ^3^Department of Sports, University of Science and Technology Beijing, Beijing, China

**Keywords:** MCI, EEG, exercise therapy, efficiency enhancement, combination

## Introduction

As the global population aging evolves, the proportion of elderly people with cognitive impairment is also increasing. Mild Cognitive Impairment (MCI) is the intermediate stage between normal aging and early dementia, accompanied by a decline in some cognitive functions (Petersen, 2004; Albert et al., 2011). Since approximately forty six percent of patients develop dementia within three years (Pal et al., 2018), it is urgent to find an effective treatment to delay the disease progression. However, there is no clear standard for drug treatment of MCI patients at present (Teixeira et al., 2012; Chen et al., 2023). For this reason, non-pharmacological therapies were gradually used to delay cognitive decline in the MCI patients (DCunha et al., 2018). The rate of glucose metabolism in the brain is one of the factors that improve cognitive ability. The slower the metabolism, the more severe the cognitive impairment. The literature available emphasizes moderate exercise can accelerate the rate of glucose metabolism in the brain, thereby improving the cognitive ability (Zhao and Xu, 2021). Moreover, the MCI patients can benefit from the recovery of physical activity, such as executive, memory and independent functions (Nuzum et al., 2020).

In recent years, with the rapid development of neuroscience and medicine, some new non-pharmacological therapies of MCI have been proposed, like dual-task training (DTT) (Norouzi et al., 2019; Oliva et al., 2020; Kannan and Bhatt, 2021), resistance exercise (Hong et al., 2018), fall-resistance training (Bhatt et al., 2012), etc. Furthermore, electroencephalography (EEG)-based exercise therapies, such as open-loop EEG-based exercise therapy (Amjad et al., 2019b; Liao et al., 2019) and closed-loop EEG-based exercise therapy (Cisotto et al., 2021), have shown great potential for clinical application regarding MCI. This paper summarizes and analyzes the relevant literature on non-pharmacological therapies for MCI patients, including exercise and EEG-based exercise ones. In light of prior study, we are also concerned that whether EEG signals really enhance the efficacy of exercise therapy, and finally propose our own opinions about development trends of EEG-based exercise therapy for MCI patients, hoping to provide useful suggestions in the future.

## Exercise therapy

Exercise improve cognitive abilities by altering the plasticity of neuronal cells (Kempermann et al., 2010; Pedroso et al., 2021), and increase the levels of clusterin (CLU) protein in plasma, which target the cerebrovasculature and benefit the brain (De Miguel et al., 2021). In terms of cognition, exercise therapy modes can be further categorized according to the required inherent energy type into: physical activities and motor activities (Voelcker-Rehage and Niemann, 2013). As will be shown later, both modes are effective for MCI patients.

Physical training activities include but not limited to aerobic exercise, strength training, etc. Treadmill rehabilitation exercise as a kind of aerobic exercise enhances furin activity induced by low iron, promotes α-secretase-dependent processing of amyloid precursor protein (APP), and reduces cognitive decline and amyloid-β-induced neuronal cell death. This provides relevant evidence for iron-targeted treatment based on treadmill rehabilitation training (Choi et al., 2021). Moreover, water sport may be more effective in MCI exercise therapy. The combination of water sports and cognitive training (CT) was compared with the one of land sports and CT for MCI patients in Campbell et al. (2023). It was found that the former had better effects on cognitive recovery of elderly MCI patients. This may be attributed to the greater cognitive training load in the water environment and the reduced impact of gravity, allowing patients to receive higher intensity training. The improvement of cognitive function is related to the intensity of exercise therapy (Yang et al., 2020). Immersing part of the body in water enhances cerebral blood flow (Carter et al., 2014), which is related to better cognitive abilities (Barnes, 2015). Although a recent meta-analysis showed that physical training has a little impact on cognitive performance, long-term physical training still plays an important role in delaying dementia (Iso-Markku et al., 2024). Thus, we should seek more effective forms of exercise.

Motor activities consist of balance, coordination, and involve high neuromuscular demands. For instance, some researchers artificially created slip hazards using block of slips in resistance training for older adults, and proved that the balance ability of elderly participants improved and could be sustained for 6 months or even longer after training (Bhatt et al., 2012). Additionally, significant increment of Montreal Cognitive Assessment (MoCA) scores after balance training demonstrated that it can also improve cognitive abilities of the elderly (Greblo Jurakic et al., 2017).

The combination of physical training activities and motor training activities will bring a new hope in MCI non-pharmacological therapy. Dance is an aerobic exercise, and requires the individuals to achieve specific postures and maintain balance. Hence, it could be considered as an exercise mode consisted of both physical training activities and motor ones. For instance, the impact of international dance on the cognitive abilities of MCI patients has been explored by Lazarou et al. (2017). 63 MCI patients participated into a 10-month international social dance training program. Neuropsychological assessment methods were utilized to compare the cognitive performance of the patients before and after the experiment. It was found that the neuropsychological test performance of most MCI patients improved, proving that the international social dance training method can enhance the cognitive abilities of MCI patients. Besides, a novel rhythm movement sequence (RMS) was designed to evaluate the effects of age-related declines in motor and cognitive functions on the ability of accurately regulating gait spatiotemporal characteristics (Rosenberg et al., 2023). The RMS represents an early step in personalized dance treatment.

Overall, physical training affects neuroplasticity and cognition via improvement in cardiovascular fitness, whereas motor training is task-specific in the enhancement of brain neuroplasticity and cognition (Netz, 2019). Having understood the mechanism of how these two modes affect cognition, we anticipate that the combined exercise mode will emerges as the predominant approach for enhancing cognitive abilities in MCI patients in the future. Even though new exercise therapies combined two modes showed great potential, some non-negligible limitations are still needed to be solved. For instance, personalized treatment plans and standardized protocols for dance training are not yet fully developed (Menezes et al., 2022; Rosenberg et al., 2023). The ideal intensity, frequency, and suitable dance styles for dance training are still uncertain (Bracco et al., 2021). Thus, it is of great importance to find an innovative strategy to evaluate and guide the process of therapeutic exercise.

## Combination of EEG and exercise therapy

EEG is one of the most common tools for recording brain activity (Sebastián-Romagosa et al., 2020). It is considered as a potential indicator for detecting changes in cognitive abilities and neurodegenerative diseases (Styliadis et al., 2015). Analysis of EEG signals offers a more objective assessment of treatment effects by examining physiological changes in patients' brains. The combined use of EEG and exercise therapy provides a novel conception, which can be applied for evaluation and guidance of rehabilitation in individuals with MCI. At present, there are two primary approaches that combine EEG with exercise therapy: open-loop EEG-based and closed-loop EEG-based ones.

For the open-loop EEG exercise therapy, EEG provides a deeper understanding of the mechanism regarding exercise intervention (Amjad et al., 2019a) and compensates for the limited accuracy of identify of MCI (Snyder et al., 2011). The decline in cognitive function of MCI patients usually exhibits increased delta and theta band power, decreased alpha and beta band power, reduced EEG complexity, and perturbed EEG synchrony (Prichep et al., 2006; Baker et al., 2008; Dauwels et al., 2010; Fauzan and Amran, 2015). Long-term exercise training for 6-12 weeks resulted in a decrease in the delta and theta band power, an increase in the beta and alpha band power, as well as an increase in the complexity and connectivity of the EEG (Pedroso et al., 2021). These changes suggest that exercise improves cortical activity and cognition in MCI patients. Furthermore, a 6-week Xbox 360 Kinect exercise cognitive game training treatment on MCI patients was conducted in Amjad et al. (2019b). The EEG analysis results showed improvement in the alpha, theta, and beta2 bands, and an increase in approximate entropy (ApEn) after training. As a result, the proposed Xbox 360 Kinect games indicated beneficial effects on MCI patients after the intervention. A 12-week resistance training therapy was proposed and the training effects were evaluated according to scales and EEG features (Hong et al., 2018). While there was a subtle improvement in the K-MoCA scores of MCI patients, a significant decrease in the theta band power at F3 (left frontal) and a significant increase in the alpha band power at T5 (left temporal) of the International 10–20 system for the MCI exercise group were observed compared to the MCI control one. The findings suggest that EEG has a higher resolution of cognitive assessment for MCI patients compared to K-MoCA.

MCI patients often experience a decline in physical motor control abilities (Montero-Odasso et al., 2012), which results in a higher risk of falls and injuries during exercise therapy. Open-loop EEG-based exercise therapy has shown outstanding performance on assessment as mentioned above, but they are often lack of responsive outcomes to prevent the patients from suffering various risks. By contrast, closed-loop EEG-based exercise therapy utilizes the feedback in time. In this case, EEG serves a dual purpose in clinical settings: Firstly, it provides feedback on physiological changes in the brain, such as levels of attention (Hamadicharef et al., 2009), which is a crucial factor in predicting the risk of falls in MCI patients (Holtzer et al., 2007); Secondly, the collected EEG signals can be used for neuro-feedback training (NFT), with better outcomes compared to the one without NFT (Jirayucharoensak et al., 2019). Therefore, the therapists would deliver a more secure and effective rehabilitation strategy for MCI patients with closed-loop EEG.

In conclusion, with the closed-loop EEG, it is easy to generate neuro-feedback stimulation which may serve as the main reason of an enhanced exercise therapy effectiveness for MCI patients. However, the closed-loop EEG-based exercise therapy for MCI is still a relatively new field of study, and there are limited researches and applications in this field.

## Present issues and future research perspectives

Relevant research achievements demonstrate that EEG significantly improve the effectiveness of exercise therapy, especially for the closed-loop EEG. With the closed-loop EEG, it is possible to provide personalized treatment plans for each MCI patient. The EEG-based therapy usually consists of the following steps: EEG signals acquisition, pre-processing, evaluation or feedback. As is known to all, EEG signal is sensitive and easily contaminated with unwanted components such as noises and artifacts caused by eye blinks, muscle movements and etc. The application of EEG during exercise therapy poses new challenges to signal acquisition and processing. To realize such personalized adaptive therapy, numerous issues need to be addressed as below.

It is needed to seek a non-vigorous exercise mode that minimizes the noises and artifacts during EEG signals acquisition. Exercise of lower limbs like walking and cycling with less head movement would be more appropriate. For instance, an EEG attention pedal training with multimodal feedback for post-stroke cognitive impairment patients was designed by Yuan et al. (2021). The closed-loop EEG training demonstrated a significant improvement compared to dual-task training without EEG, and it holds potential for future use in the therapy for MCI patients.

Behaviors such as blinking and muscle bursts usually cause high-amplitude EEG artifacts. Currently, the mainstream artifact removal methods include Principal Component Analysis (PCA) and Independent Component Analysis (ICA). Besides, wireless EEG or EEG-EMG intergrated devices are another acceptable solution for artifacts removal (Bigliassi et al., 2021). However, EEG noises and artifacts removal during complex exercise training still faces significant challenges (Chen et al., 2023). We advocate that researchers should continue to improve the performance of movement-induced EEG artifacts removal methods, especially for the closed-loop EEG-based approach.

According to Hosang et al. (2022), highly integrated and small-sized equipment ensures the quality of EEG signal collection during exercise. The level of integration of the current treatment devices is relatively low, requiring patients to go for regular exercise sessions under the supervision of a trained therapist in medical institutions like rehabilitation center. Designing a highly integrated training system could reduce the burden on both patients and therapists (Holtzer et al., 2007; Cisotto et al., 2021), let patients get trained at home, and would greatly ensure the timeliness of treatment.

In addition to the above issues, even though we recommend the use of closed-loop EEG-based exercise therapy, the sample size of the experiments in the literature is still relatively small. The lack of sufficient experimental data would result in incorrect evaluation and judgment of the practical effectiveness of above treatments. The need to examine the effectiveness of the combination of NFT and exergames (also called active-play video games) was also emphasized (Jirayucharoensak et al., 2019). Therefore, it is necessary to increase the experimental data for some existing closed-loop EEG-based exercise therapies.

Moreover, although exercise influences performance in the several cognitive domains (Zhou et al., 2020), it lacks the intensive training for particular cognitive domain. Recent systematic review and meta-analysis also found only weak association between physical activity and cognition (Iso-Markku et al., 2024), so it is of necessity to compensate for the shortcomings of single physical training. Motor and cognitive dual-task training is effective on the cognitive rehabilitation for MCI patients (Styliadis et al., 2015; Oliva et al., 2020), and it performs better than single-task training (Kannan and Bhatt, 2021). Different exercise modes also show diverse performance on DTT for MCI therapy (Campbell et al., 2023). In light of above, we recommend taking DTT in future therapy for MCI.

## Conclusion

In summary, while the standard of drug treatment for MCI patients is unclear, the innovative EEG-based exercise therapies offer new hope. We suggest collecting EEG activities of the patients during the exercise therapy to adjust and monitor the progress of the rehabilitation. Our ultimate goal is to develop a closed-loop EEG-based exercise therapy system. But it must overcome significant issues. Future research perspectives should deal with the following topics:(1) Seeking suitable EEG exercise mode, (2) Exploring EEG motion artifacts removal methods, (3) Creating a highly integrated EEG-based exercise training system, (4) Aquiring sufficient experimental data, (5) Proposing motor and cognitive dual-task training. From our vision, further in-depth researches in this field would give the optimal treatment for MCI patients.

## Author contributions

XW: Project administration, Supervision, Writing – review & editing. YZ: Writing – original draft, Writing – review & editing. TL: Writing – review & editing. DL: Writing – review & editing. HY: Writing – review & editing. DW: Writing – review & editing, Supervision.
